# Salt Ion Accumulation in Bipolar Membranes Limits the Maximum Rate of Neutralization

**DOI:** 10.1021/acsami.5c08661

**Published:** 2025-07-29

**Authors:** Pavel A. Loktionov, Erik M. Kelder, David A. Vermaas

**Affiliations:** † Department of Chemical Engineering, Delft University of Technology, Van der Maasweg 9, Delft 2629HZ, The Netherlands; ‡ Department of Radiation Science and Technology, Delft University of Technology, Mekelweg 15, Delft 2629 JB, The Netherlands

**Keywords:** bipolar membranes, forward bias, ion exchange, mass transport, neutralization reaction

## Abstract

Bipolar membranes (BPMs) emerge as a valuable component in novel energy conversion devices utilizing a water-splitting reaction within BPMs. However, the opposite process, proton and hydroxide recombination (forward bias), remains challenging to control due to its strong dependence on the electrolyte composition. Even minor contamination of acid and base solutions by salt can significantly compromise the BPM performance. This study examines the impact of salt contamination on the BPM performance under forward bias. The results reveal that, during neutralization, salt ions accumulate near the BPM junction, hindering H^+^ and OH^–^ transport toward the catalytic interface. Notably, the anion-exchange layer exhibits a high sensitivity to salt contamination in the base solution, with active site swapping between OH^–^ and anions emerging as the rate-determining step. The extent of this transport limitation depends on the acid/base-to-salt ratio. To address this issue, mitigation strategies are explored, including asymmetric BPMs. Reducing the thickness of the anion-exchange layer significantly enhances OH^–^ mobility, thereby increasing the limiting current density of neutralization in salt-contaminated electrolytes. These insights offer a deeper understanding of mass-transport limitations in BPMs and highlight pathways to optimize performance in energy conversion applications.

## Graphical abstract

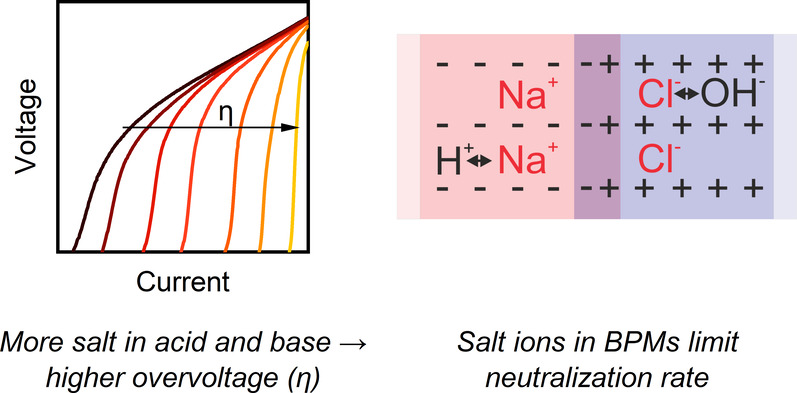

## 1 Introduction

Electrochemical energy conversion represents a pivotal pathway for advancing the global energy transition, providing sustainable solutions for energy storage, fuel production, and environmental remediation. Within this framework, bipolar membranes (BPMs) have emerged as a critical component offering the unique ability to independently control the ionic environment of cathodic and anodic half-reactions in electrochemical cells. This distinctive property not only improves the efficiency and selectivity of redox reactions but also broadens the operational flexibility of the electrochemical systems.

BPMs are composed of cation- and anion-exchange layers (CEL and AEL, respectively) pressed against each other with a thin layer of a water dissociation catalyst.^1–3^ Typically, CELs are based on sulfonated polymers, while AELs commonly contain quaternary ammonium-functionalized polymers.^1,2^ Depending on the polarity of the applied current, BPMs can either split water into protons and hydroxide ions (reverse bias mode) or neutralize H^+^ and OH^–^, forming water (forward bias). To date, the most widely used catalysts for water dissociation in BPMs are graphene oxide and metal oxides,^1^ particularly TiO_2_ and SnO_2_, with the latter demonstrating a superior performance.^4^ Although the same catalyst can, in principle, operate under both reverse and forward bias, the optimal catalyst properties differ significantly for each mode.^5^ For example, water dissociation benefits from an electrically conductive catalyst that concentrates the electric field to improve voltage efficiency, whereas water recombination requires a thin catalytic interface to minimize transport limitations.^5^

Until recently, BPMs were mainly used under reverse bias for the production and recovery of acids and bases,^6^ pH management in various industrial processes,^7^ CO_2_ capture using pH-swing methods,^8–10^ water electrolysis,^11–14^ and pH control in CO_2_ electrolysis.^15–17^ Alternatively, electrochemical technologies have been proposed using BPMs in forward-bias mode where water recombination occurs. These technologies include hybrid fuel cells,^18–21^ acid–base flow batteries (ABFBs),^22–25^ CO_2_ electrolyzers,^16,26–29^ and energy-harvesting devices.^30^ However, because commercial BPMs have primarily been optimized for water dissociation, forward-bias operation often encounters poorly understood performance limitations.

One of the main hurdles in developing forward-bias-based devices is the complexity of water recombination when using electrolytes contaminated with salt ions. The presence of salt in the acid or base solution results from salt-ion crossover through the cell membranes (mainly, BPMs)^31,32^ and can greatly affect the overall performance of a BPM-based device.^33,34^ While recent studies have explored the accumulation of weak bases in BPMs, typically under low pH gradients, as in CO_2_ electrolyzers,^35–37^ the accumulation of cations and anions in BPMs operating with strongly acidic and alkaline electrolytes, as used in ABFBs,^38,39^ remains poorly understood and lacks a comprehensive mechanistic description.

In this study, we explore forward-bias operation in BPMs, using either pure acid and base solutions or electrolytes contaminated with the corresponding salt. We found that even a minor fraction of salt ions in the electrolyte migrates inside the membrane layers, hindering H^+^/OH^–^ transport toward the catalyst interface and reducing the limiting current by severalfold. Using the data for different acid/base-to-salt ratios, we describe the underlying mechanism of this phenomenon. We suggest that the maximum rate of neutralization depends on diffusion of H^+^ and OH^–^ through a layer of salt ions. Moreover, we analyze salt selection and membrane thickness as possible strategies to mitigate this issue. We believe that our findings lay the foundation for a better understanding of this phenomenon and will facilitate the development of more efficient energy conversion devices.

## 2 Experimental Section

### 2.1 Chemicals and Materials

All of the chemicals used in the study were ACS-grade or higher and were used as-received without any additional purification.

The BPMs used in the study were Fumasep FBM^40^ and tailor-made BPMs. Apart from a BPM of interest, we used cation-exchange Fumasep FKD-PK-75 in a six-compartment cell (see the description below). Fumasep FBM and Fumasep FM-FKD-PK-75 were presoaked in 0.5 M NaCl overnight prior to the experiments. Custom-built BPMs were obtained from Fumasep PFSA-D50 or Nafion 211, 212, 115, or 117 (CEL) and Piperion 15, 20, 40, 60, or 80 (AEL). The layers were used as-received and soaked in deionized water prior to membrane assembly. Custom-built BPMs were also presoaked in deionized water before the experiments. Additional details on the membrane preparation and preconditioning are given below.

### 2.2 Experimental Setup

For all of the electrochemical experiments on membranes, we used a six-compartment electrochemical cell. All of the experiments were performed at 22 ± 1 °C unless otherwise stated. Two central compartments were filled with “acid” and “base” solutions and separated by the investigated membrane. Two reference electrodes (Ag/AgCl and 3 M KCl) were connected to the acid and base compartments via a Haber-Luggin capillary from both sides of the membrane (the distance between the capillary tip and a membrane was 2 mm). These two compartments were separated by two cation-exchange membranes from the next two compartments, which were filled with a 0.5 M phosphate buffer solution (pH = 7). All compartments were arranged between two electrode chambers, which were separated from the adjacent compartments by cation-exchange membranes and filled with a 0.25 M Na_2_SO_4_ solution. The cell was supplied with solutions via peristaltic pumps for continuous circulation of the electrolytes through the corresponding compartments. More details on this six-compartment cell have been published previously.^41,42^

### 2.3 Polarization Curves for Reverse and Forward Biases

Prior to each experiment, the cell was flushed with distilled water. After the water was removed from all of the compartments, a membrane sample (9.62 cm^2^) was placed between the acid and base compartments, and the cell was filled with a given series of electrolytes. The volume of the acid and base electrolytes was 0.25 L each, and the volumes of the phosphate buffer solution and sodium sulfate solution were 0.5 L each. Fresh membrane samples were used for each new experiment.

The concentrations of H^+^ in an acid solution and OH^–^ in a base solution were the same, while their maximum concentration was 0.5 M. The ionic strength was kept constant for all cases, while the acid/base-to-salt ratio was different. See Table 1 for further information on the electrolyte solution facing CEL and AEL.

**Table 1:** Composition of Acid and Base Solutions for the Case of NaCl-Based Electrolytes, Depending on the Degree of Salt Content

		CEL side		AEL side	
salt content (%)	acid/base-to-salt ratio	[HCl] (M)	[NaCl] (M)	[NaOH] (M)	[NaCl] (M)
0		0.5	0	0.5	0
25	1:0.167	0.375	0.0625	0.375	0.0625
50	1:0.5	0.25	0.125	0.25	0.125
75	1:1.5	0.125	0.1875	0.125	0.1875
100		0	0.25	0	0.25

Besides the above-mentioned solutions, other electrolyte series were used either to show the effect of the salt content on the membrane performance or to reveal the nature of the membrane polarization. For example, 0.25 M HCl and NaOH solutions were used with the addition of 0.125 M NaCl in acid and/or base or without it.

Electrochemical measurements of the membranes were conducted using an Autolab PGSTAT302N (Metrohm, Switzerland) as follows. First, the membrane sample was rested for 10 min or until a stable open-circuit voltage (OCV) was obtained (dU dt^–1^ < 1 mV h^–1^). Then we measured a linear sweep of the current for reverse bias (sweep rate of 50 μA cm^–2^ s^–1^ from 0 to 100 mA cm^–2^ or until reaching voltage overload). Next, we recorded the OCV for 10 min or until stabilization of the signal (dU dt^–1^ < 1 mV h^–1^) and measured the linear sweep of the current for the forward-bias mode (sweep rate of 50 μA cm^–2^ s^–1^ from 0 mA cm^–2^ until reaching 0 V). Stationary values of the potential drop across a BPM, which were measured after reverse bias, were used as the OCV of the membrane.

The apparent selectivity of the membrane was calculated as a ratio between the measured and calculated (using the Nernst equation)^43^ values of the OCV. To assess the limiting current density under forward bias, we extracted the current value at 0 V on a polarization curve.

To assess the reproducibility of the results, at least two samples of the membrane (from two different batches) were used in the case of NaCl-based electrolytes. Where appropriate, we provided mean values of measured membrane metrics along with the corresponding standard deviation (calculated using a confidence level of 95%).

### 2.4 Electrochemical Impedance Spectroscopy

To reveal the membrane resistance breakdown, we used electrochemical impedance spectroscopy. We recorded impedance spectra of BPMs in potentiostatic mode at a given voltage with an amplitude of 10 mV in the frequency range from 100 kHz to 5 mHz. Spectra were recorded either versus OCV or as a voltage under reverse or forward bias, which corresponds to +20, +10, +5, 0, −1, −2, −3, −4, or −5 mA cm^–2^.

Following earlier studies on electrochemical impedance spectroscopy of BPMs,^44,45^ all of the spectra were treated in *Nova* software using a modified Randles circuit [R­(RC)­(RC)] (Figure 1). The spectra were used to determine the resistance of electrolytes and membrane layers (*R*_s_), resistance of water dissociation (*R*_WD_) or neutralization (*R*_N_), specific capacity of the membrane interface (*C*_1_), resistance of ionic blockade (*R*_IB_),^37^ and the corresponding capacitance (*C*_2_). For more information about the nature of these components, see the main text.

**Figure 1:**
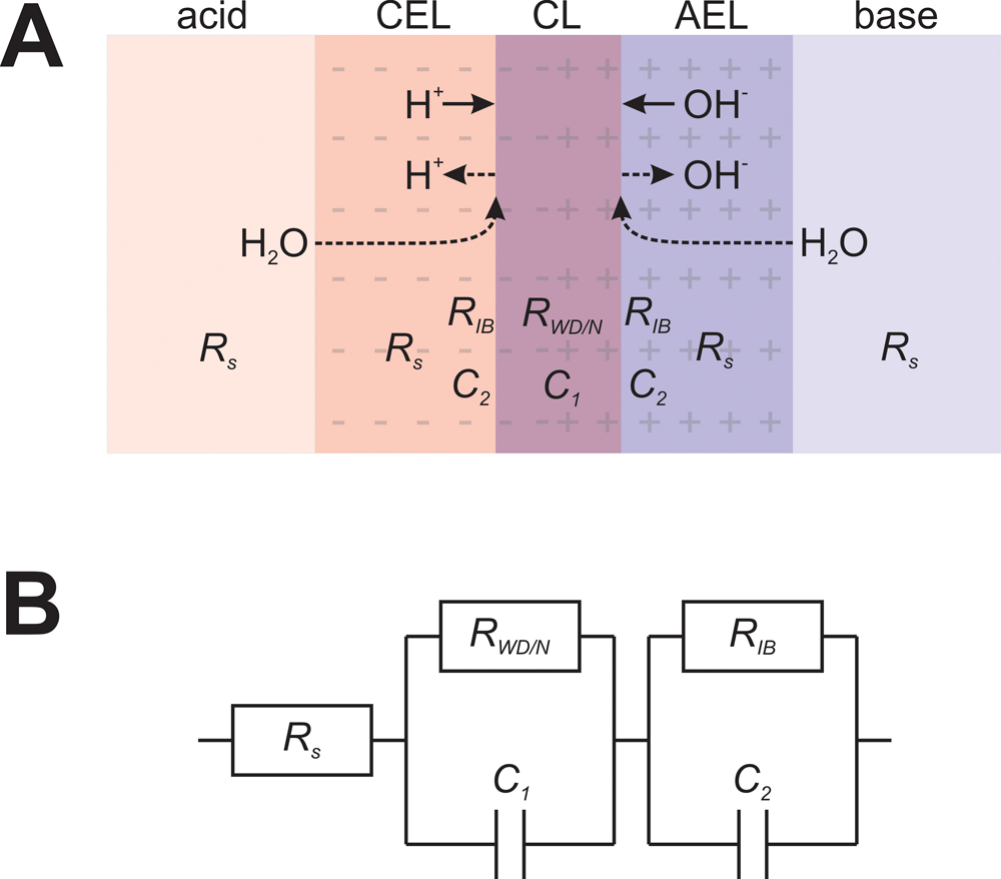
Scheme of a BPM (A) under reverse (dashed lines) and forward (solid lines) biases, with an indication of individual components of resistance and the corresponding equivalent circuit (B) used to fit the measured spectra.

### 2.5 Preparation of Asymmetric BPMs

In addition to Fumasep FBM, asymmetric custom-built membranes were used to test hypotheses related to salt accumulation. These membranes were prepared using a common approach: assembling a BPM from individual ion-exchange layers with a catalyst layer in-between (see an example in a recent study^46^). For preparation of the asymmetric BPMs, a CEL (Fumatech PFSA-D50-U or Nafion 211, 212, 115, and 117) and an AEL (reinforced Piperion 15 or nonreinforced Piperion 20, 40, 60, or 80) were used as received with the layer of graphene oxide (GOx) between them. The underlying mechanism of membrane assembly is the chemical interaction between the perfluorosulfonic acid-based CEL and poly­(arylenepiperidinium)-based AEL, with a thin catalyst layer in-between. This structure forms an efficient catalytic junction for water splitting and neutralization.^46^ Although the CEL and AEL have different polymer backbones, and therefore the interfacial adhesion is not ideal,^47,48^ it was sufficient for evaluating the proposed hypotheses on ion transport within the membrane layers. It is also important to note that, in asymmetric membranes, strong bonding between the layers is even more critical because differences in swelling between the layers pose an additional risk to the mechanical stability of the catalytic junction.^46,49,50^

To prepare the catalyst ink, graphene oxide paste (Graphene Supermarket) was diluted by deionized water from 30 to 10 g L^–1^ and sonicated in an ultrasonic bath for 10 min. To obtain a catalyst ink, 2 mL of the GOx suspension (10 g L^–1^), 17 mL of a 1:1 H_2_O/isopropyl alcohol mixture, and 1 mL of a Nafion D520 dispersion were mixed and sonicated in an ultrasonic bath for 10 min prior to membrane coating.

A custom-built spray-coating setup (see details in the authors’ previous paper)^51^ was used to apply a GOx catalyst to cation-exchange membranes. A sample of the cation-exchange membrane was taped to a heating plate (130 °C) of the setup, and the catalyst ink was spray-coated using a CNC platform in a “serpentine” pattern (the duration of the coating is less than 1 min). The coated membrane was then placed in an oven at 100 °C for 10 min for complete evaporation of the solvents. Due to the small membrane size and low solid content of the ink, the GOx loading was estimated based on consumption of the catalyst ink. Although previous spray-coating trials with other inks showed significant ink losses (20–30%), these losses were not accounted for here because the catalyst performance was not the primary focus of this study. Prior to the membrane preparation, the anion-exchange membrane was soaked in deionized water and the coated cation-exchange membrane was wetted with a few droplets of deionized water from the noncoated side of the membrane. The membrane assembly process was performed on the glass plate and involved placing the wet anion-exchange membrane on top of the coated side of the wet cation-exchange membrane. Right after that, the layers were manually pressed together between glass-plated and gloved fingers. Any remaining air pockets and liquid between the layers were squeezed outside of the membrane using a paper towel. Finally, the membranes were rewetted in deionized water and tested in the six-compartment cell right after preparation.

### 2.6 Stress Tests of BPMs under Forward Bias

To assess the structural integrity and potential catalyst degradation caused by salt-ion accumulation inside the membrane, stress tests were performed on BPMs for 1 h at −50 mA cm^–2^ using different acid/base-to-salt ratios (1:0, 1:0.5, and 0:1). Before and after each stress test, we measured the polarization curves and impedance spectra under reverse bias at 20 mA cm^–2^. On the basis of the change in *R*_WD_, we analyzed the membrane degradation.

## 3 Results and Discussion

### 3.1 Overvoltage in BPMs under Forward Bias

In line with previous works,^37,38^ a forward bias of BPMs is greatly affected by the presence of salt in acid and base solutions (Figure 2A). This issue specifically arises from acid and base cross-contamination because the earlier study showed that acid and base crossing into the salt compartment simply recombine to form salt.^52^ After 0.125 M NaCl is introduced into both 0.25 M HCl and NaOH (i.e., for the case of bilateral contamination), the OCV of the membrane decreases from 724 to 716 mV, while the maximum rate of neutralization, i.e., limiting current density, drops from over 53 to 10.6 mA cm^–2^.

**Figure 2:**
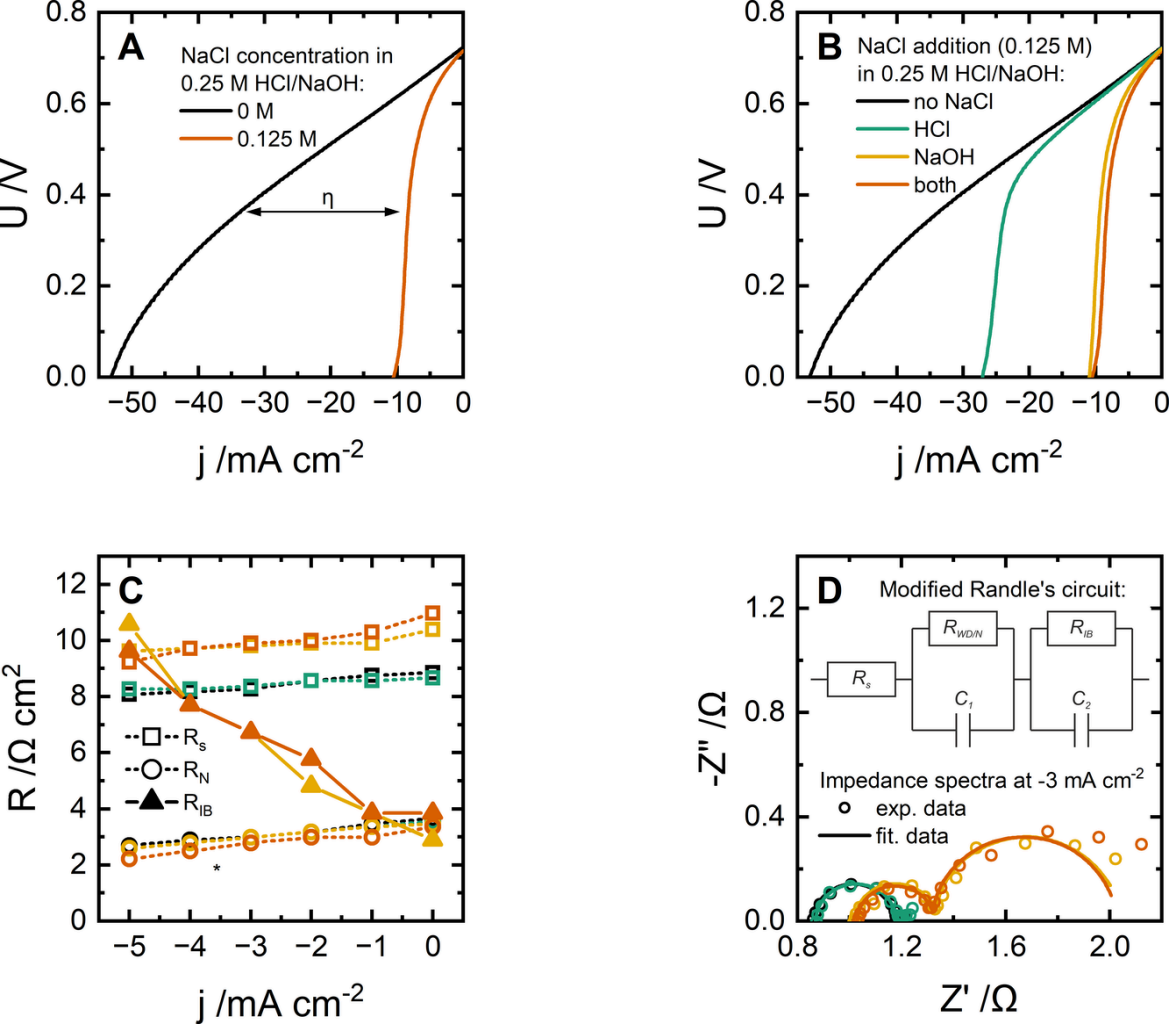
Forward bias of Fumasep FBM, which uses either “pure” or “contaminated” electrolytes (A) or uses NaCl in acid or base solutions (B), data on electrochemical impedance spectroscopy (C), and example of impedance spectra with the equivalent circuit used (D). Lines on scattered graphs are just a guide for the eye; *on panel C, the shapes for the “no NaCl” and “HCl” cases overlap.

Importantly, we observe that the CEL and AEL of the membrane are not equally sensitive to electrolyte contamination by salt (Figure 2B). Polarization curves of the membrane with NaCl added to either HCl or NaOH (unilateral contamination) suggest that the maximum rate of neutralization is more strongly affected by the presence of Cl^–^ in the AEL rather than by Na^+^ appearing in the CEL.

Electrochemical impedance spectroscopy (Figure 2C) provides further insight into this phenomenon. We use a *R*_s_[*R*_N_/*C*_1_]­[*R*_IB_/*C*_2_] equivalent circuit to describe the membrane behavior (Figure 1), following reported models in earlier studies.^44,45^ The first component, *R*_s_, represents the resistance of the membrane layers and the electrolyte layers between the membrane and reference electrode probes. Next, an RC element models the neutralization reaction, where *R*_N_ describes the kinetics of this reaction and the double-layer capacitance *C*_1_ at the interface where this occurs. Finally, we introduce an additional RC element related to the diffusion of species near the membrane interface, which is significant only when using “contaminated” electrolytes (Figure 2D). We define this resistance as *R*_IB_ because it behaves as an ionic blockade for H^+^ and OH^–^. Further details about the nature of this resistance are given below. Therefore, to be recombined, protons and hydroxides migrate toward the membrane junction, where they sequentially pass through the layer of salt ions; i.e., migration and diffusion of H^+^ and OH^–^ occur in series, as suggested by the equivalent circuit (Figure 1).

When the current density magnitude is increased (Figure 2C), both *R*_s_ and *R*_N_ slightly decrease. Meanwhile, *R*_N_ is more than 3 times lower than *R*_s_, and the latter is sensitive to the presence of NaCl in the base solution: the resistance *R*_s_ increases when NaCl is added to the NaOH solution, which can be explained by the lower membrane conductivity in the Cl^–^ form compared to its OH^–^ form. In contrast, the CEL appears unaffected by Na^+^ contamination in the acid solution, possibly due to its high affinity to H^+^, which keeps the Na^+^ concentration inside the CEL relatively low. Importantly, *R*_IB_, which appears in the impedance spectra at low frequencies (from 1 Hz to 5 mHz) when the solutions are contaminated (Figure 2D), has a nonzero value at 0 mA cm^–2^ and increases with the neutralization rate. At just −5 mA cm^–2^, *R*_IB_ becomes the dominant contribution to the overall resistance, which we identify as the primary cause of the low limiting current density in the polarization curves for contaminated acid/base (Figure 2A,B).

Given the strong dependence of *R*_IB_ on the current density and its characteristic frequency range, we suggest that this resistance reflects mass-transport limitations for H^+^ and/or OH^–^ ions attempting to pass through Na^+^/Cl^–^-rich membrane layers near the junction. The independence of the limiting current density from the electrolyte flow rate (Figure S1A) further supports that the transport-limiting stage occurs within the bulk membrane rather than in the bulk electrolyte. Moreover, the maximum rate of neutralization depends not on the total concentration of the electrolytes but rather on the acid/base-to-salt ratio (Figure S1B). We suggest that, under limiting current density, the amount of salt ions accumulated in the membrane layers depends on the transport properties of Na^+^ and Cl^–^ ions in the membrane. Furthermore, we suggest that the concentration of salt ions in the membrane affects the transport of H^+^ and OH^–^ ions toward the junction.

Next, we highlight that *R*_IB_ is nonzero even when there is no current applied to the membrane, indicating that even a small initial amount of Na^+^ and/or Cl^–^ ions impairs the transport of H^+^ and OH^–^ toward the interface between the layers. The effect of salt-ion accumulation in the membrane layers is also evident under reverse bias (Figure S2). We suggest that salt ions hinder the transport of H^+^/OH^–^ from the catalyst interface toward the bulk electrolyte, as indicated by higher energy consumption for water dissociation at *j* < 20 mA cm^–2^ (Figure S2A). At higher current densities, Na^+^ and Cl^–^ ions are effectively expelled from the membrane (or at least from the catalytic interface; see the discussion in the following sections), as shown by negligible *R*_IB_ at 20 mA cm^–2^ (Figure S2B,C).

### 3.2 Contamination-Dependent Overvoltage in BPMs

To further confirm the distinct transport behavior of H^+^/OH^–^ in the case of “contaminated” electrolytes, we present polarization curves and electrochemical impedance spectra for bilaterally contaminated acid and base with different acid/base-to-salt ratios (Figure 3). When shifting from pure 0.5 M HCl and NaOH to acid/base-to-salt ratios of 1:0.167, 1:0.5, or 1:1.5, the limiting current density decreases from 53 to 4.2 mA cm^–2^ (Figure 3A). Impedance spectra (Figures 3 and S3) reveal a strong dependence of *R*_IB_ on the acid/base-to-salt ratio (Figure 3B): at −3 mA cm^–2^, *R*_IB_ changes from <0.2 to 2.5 and 6.7 Ω cm^2^ for the ratios of 1:0, 1:0.167, and 1:0.5, respectively. The impedance spectrum for the smallest acid/base-to-salt ratio (1:1.5) is even hundreds of Ω cm^2^ at −3 mA cm^–2^ (Figure 3B*).

**Figure 3:**
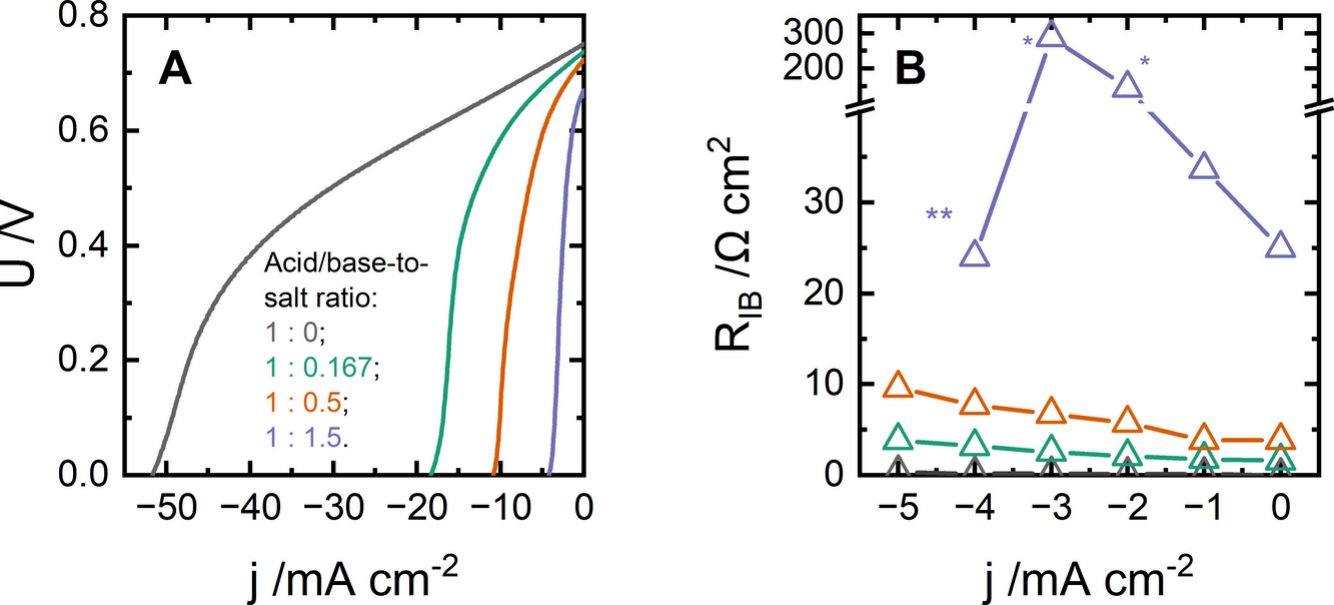
Forward bias of Fumasep FBM, which uses NaCl-based electrolytes with different acid/base-to-salt ratios (A), and dependence of *R*_IB_ on the current density for membranes, which use the mentioned electrolytes (B). Lines on scattered graphs are just a guide for the eye; note that panel B lacks a data point for −5 mA cm^–2^ for the case of the 1:1.5 acid/base-to-salt ratio. Under these conditions, the membrane potential becomes negative and the spectra becomes unstable.

The results also suggest that an increase in the neutralization rate (i.e., higher current density magnitude) aggravates the mobility of H^+^ and/or OH^–^ toward the junction. However, for the case with an acid/base-to-salt ratio of 1:1.5, *R*_IB_ decreases beyond −3 mA cm^–2^ (Figure 3B**). We hypothesize that when the salt-ion concentration in the membrane layers is sufficiently high and the membrane voltage approaches 0 V or lower, salt ions may undergo recombination near the catalytic interface. Below, we discuss this hypothesis in more detail.

### 3.3 Mechanism of Ionic Blockade in BPMs

Keeping in mind the above results, we suggest the following mechanism for the accumulation of salt ions in BPMs (Figure 4). Here, bilateral contamination is considered for simplicity. Considering complete equilibration of the membrane, when no current is applied, salt ions partially occupy the active sites of the membrane layers, and their content reflects that in the bulk of liquid electrolytes. As a result, the OCV is lower than that in the case of “pure” electrolytes. A lower OCV and nonzero *R*_IB_ at 0 mA cm^–2^ suggest that even when no current is applied, salt ions are present in the membrane layers and impair H^+^/OH^–^ mobility toward the catalytic interface. In addition to hindering the diffusion of H^+^/OH^–^, salt ions present in acid and base may eventually condense onto fixed charges within the membrane,^53–55^ thereby reducing the conductivity of the layers. This effect could partially explain the increase in *R*_s_ with a rising salt content (Figure S3A). However, at lower acid/base-to-salt ratios, this effect appears less significant compared to diffusion limitations (Figure S3A,C).

**Figure 4:**
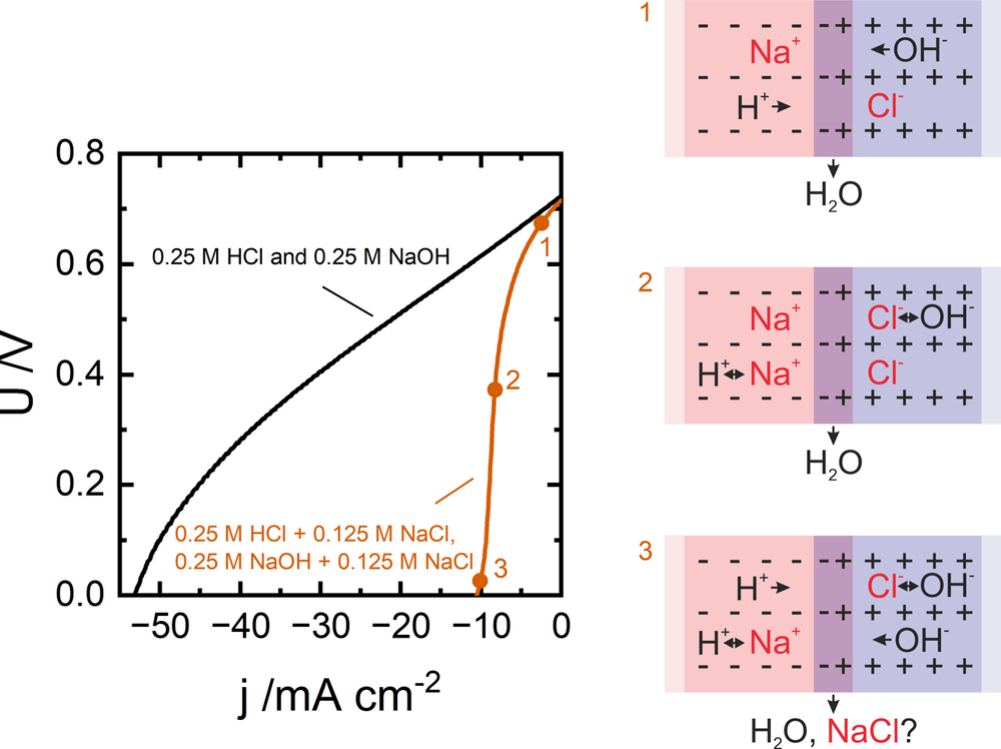
Schematic representation of salt-ion accumulation inside the layers of BPMs when forward bias is performed using salt-contaminated electrolytes. The ion-transport scheme presented here assumes symmetric composition of the membrane layers and similar transport properties of H^+^/OH^–^ in corresponding layers.

When a low current density is applied to the membrane (Figure 4, case 1), the H^+^/OH^–^ content near the junction decreases, while Na^+^/Cl^–^ moves toward the catalytic interface due to migration drag. Notably, these ions are unlikely to recombine in a given range of voltage (between p*K*_a_ of water and p*K*_a_ of NaCl), which ensures their accumulation near the catalytic interface. However, at low current densities, the migration flux of salt ions is expected to be balanced by the diffusion flux of these salt ions from the membrane returning toward the liquid electrolytes. Therefore, the Na^+^/Cl^–^ content decreases the availability of H^+^ and OH^–^ in the membrane, as highlighted by the moderate *R*_IB_, but it still allows a sufficient supply of H^+^ and OH^–^.

When going to the limiting current density (Figure 4, case 2), the H^+^/OH^–^ content in the layers further depletes, and the migration flux of Na^+^/Cl^–^ increases their concentration near the junction. The acid/base-to-salt ratio determines the transport number of salt ions and, therefore, the maximum concentration of salt ions in the membrane. At this point, the effect of ionic blockade^37^ in one or both layers becomes rate-determining. Moreover, unlike ionic blockade, salt-ion condensation is expected to decrease with increasing current density (i.e., neutralization rate), as the current governs the water activity within the membrane layers.

If we assume no Na^+^/Cl^–^ recombination, then the most likely pathway for H^+^/OH^–^ ions to reach the junction is by swapping an active site with Na^+^/Cl^–^ (see a similar mechanism proposed in a previous study).^37^ This implies that the overall rate of neutralization is determined by the diffusion coefficients of H^+^ and/or OH^–^ through the Na^+^/Cl^–^-rich regions, with salt-ion condensation playing a secondary role. To further support the dominance of diffusion limitations, we analyze the BPM performance under bilaterally contaminated conditions at both ambient and elevated temperatures (Figure 5). At 2.5 times higher temperature, the maximum neutralization rate increases by a factor of 2.3 (Figure 5A). Data on the impedance spectra (Figure 5B) indicate that this improvement is mainly due to enhanced transport of H^+^ and, more notably, OH^–^ through the respective ionic blockade layers. Moreover, assuming that this process is diffusion-controlled, we suggest that the limiting current density strongly depends on the thickness or density of the Na^+^/Cl^–^ layer. We explore this suggestion below.

**Figure 5:**
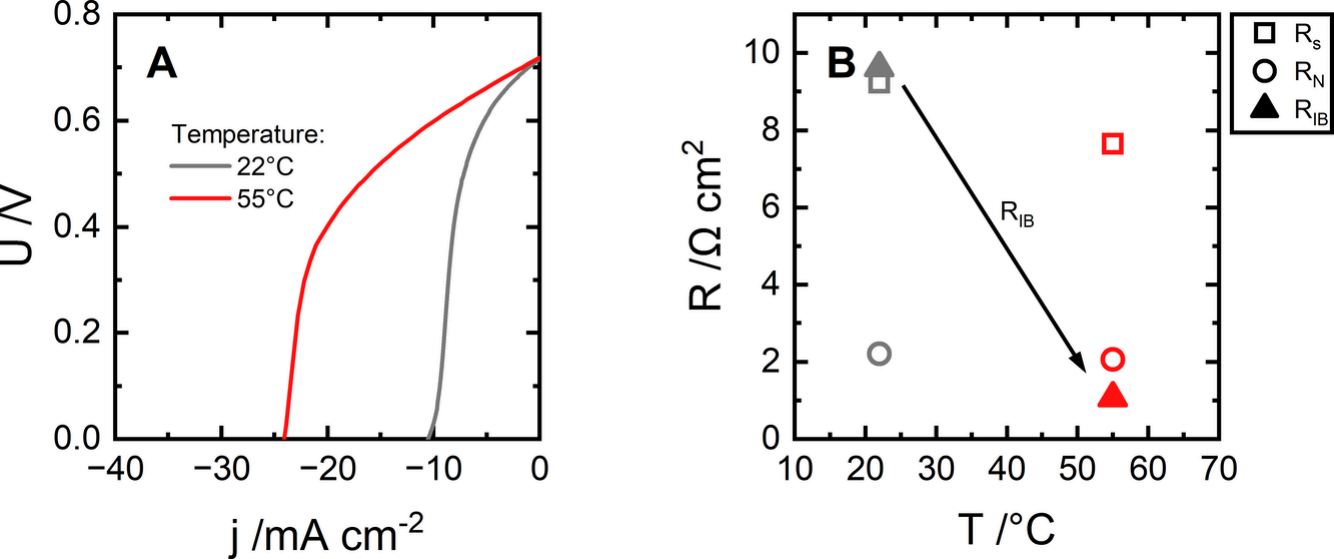
Forward-bias curves (A) and resistance breakdown (B) of Fumasep FBM at ambient (22 ± 1 °C) and elevated (55 ± 2 °C) temperatures. Electrolyte compositions: 0.25 M HCl + 0.125 M NaCl and 0.25 M NaOH + 0.125 M NaCl.

The above data on electrochemical impedance spectroscopy (Figure 3B) suggest that a further increase in membrane polarization (Figure 4, case 3) could cause the recombination of Na^+^ and Cl^–^ ions. Previous research suggests that the NaCl concentration in the junction can reach over 2 M when using a high current density,^38^ which could induce salt crystals. However, we did not observe such internal salt formation yet: there are no visible change in the membrane appearance (e.g., delamination or blisters), or any significant change in performance, even after operation at −50 mA cm^–2^ for 1 h with 0.25 M NaCl instead of acid and base solutions (Figure S4). Further research is needed to understand the degradation mechanisms of BPMs under forward bias.

### 3.4 Decrease in the Ionic Blockade Thickness Enhances the Neutralization Rate

By changing the composition of the electrolytes, one can affect the transport properties of protons and hydroxides through ionic blockades. Furthermore, we suggest that by changing the thickness of the ionic blockade, we can affect the maximum rate of neutralization. To test this hypothesis, we obtained a series of custom-built BPMs with fixed CEL thickness and various AEL thicknesses and assessed them under forward-bias conditions with the addition of salt to the base (Figure 6). We varied the thickness of the AEL layer due to its greater sensitivity to ionic blockade, although membranes with various CEL thicknesses were also tested (see below). These membranes consist of a 50 μm CEL (Fumatech PFSA-D50-U) coated with a graphene oxide catalyst (9 μg­(GOx) cm^–2^) and an AEL (Piperion) with a thickness of 15, 20, 40, 60, or 80 μm.

**Figure 6:**
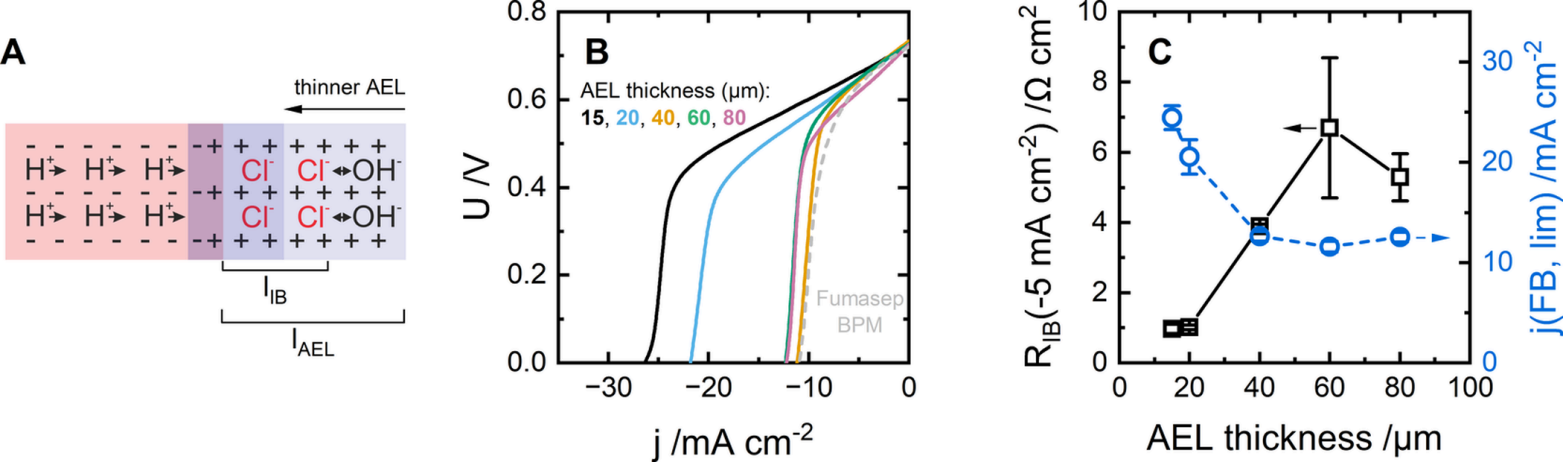
Scheme (A) and forward-bias polarization curves (B) of custom-built BPMs, which use a salt-contaminated base solution and dependences of the limiting current under forward bias and *R*_IB_ at −5 mA cm^–2^ on the AEL thickness (C). CEL is Fumatech PFSA-D50-U (50 μm), AEL is Piperion (15, 20, 40, 60, or 80 μm), and the catalyst is GOx [9 μg­(GOx) cm^–2^]. Electrolytes: 0.25 M HCl, 0.25 M NaOH + 0.125 M NaCl. Panel C includes average values of *R*_IB_ and *j*(FB,lim) with indication of standard deviation based on two membrane samples; data for Fumasep BPM are included for reference; in panel C, lines are just a guide for the eye.

According to our hypothesis of the ionic blockade, the maximum neutralization rate (of H^+^ and OH^–^) is determined by the diffusion of salt ions returning from the ionic blockade layer in the BPMs. Thus, we propose that the neutralization rate can be increased by making the AEL thinner (Figure 6A).

When using the thickest AEL (80 μm), the limiting current density is 12.6 mA cm^–2^ (Figure 6B,C). When reducing the AEL thickness from 80 to 60 and 40 μm, we see no notable change in the limiting current density and *R*_IB_. We suggest that this is because, for a given base-to-salt ratio of 1:0.5, the AEL thickness (*l*_AEL_; Figure 6A) is higher than the ionic blockade thickness (*l*_IB_). This suggests that site swapping of OH^–^ and Cl^–^ does not necessitate that Cl^–^ diffuse into the bulk electrolyte. Next, when reducing the AEL thickness to 20 or 15 μm, we observe an almost 2-fold increase in the neutralization rate. First, along with reduced *R*_IB_ (Figure 6C), this proves the hypothesis of a diffusion-limited neutralization rate. Second, it shows that, for the given conditions, *l*_IB_ is in the range from 20 to 40 μm. We conclude that reducing the AEL thickness is an effective path toward BPMs that are tolerant to the crossover of salt ions.

Notably, membranes with various CEL thicknesses do not show a significant dependence of the limiting current on the CEL thickness (Figure S5), suggesting that the impact of ionic blockade in the CEL is minimal and further constrained by water-transport limitations.

## 4 Conclusions

This study provides insights into the issue of salt-ion accumulation in BPMs under forward bias. Our results demonstrate that even small concentrations of salt ions in acid and base solutions of a BPM can severely limit the maximum neutralization rate by mass-transport limitations for H^+^/OH^–^ (i.e., ionic blockade) within the membrane layers. In this work, we propose a mechanism of this phenomenon and show that for H^+^/OH^–^ ions to be neutralized under limiting current density they must diffuse through the ionic blockade layer. We further describe that the thickness of this layer depends on the acid/base-to-salt ratio. While this study emphasizes that diffusion limitations for H^+^/OH^–^ play a dominant role in voltage losses under forward bias, salt-ion condensation within the membrane layers may also contribute. Moreover, we show that, by making the AEL thinner, we improve the neutralization rate by reducing transport limitations for H^+^/OH^–^ toward the catalytic interface. Notably, the AEL appears to be more sensitive to the presence of salt ions in the base solution than in the acid solution.

From a practical perspective, these findings suggest that, once an ABFB experiences significant salt-ion crossover between the compartments, its available power output for a discharge will be hindered by the ionic blockade in BPMs. Because the maximum neutralization rate is mainly limited by H^+^/OH^–^ transport within salt-ion-rich regions within the membrane layers, the associated voltage losses in ABFBs are expected to be dependent on the temperature. Tuning the composition of the electrolytes and BPM layers can help to make the battery more tolerant to crossover. However, future research should analyze the BPM performance in parallel with the rate of salt-ion crossover between the compartments.

By understanding the mechanism of the ionic blockade and highlighting pathways to overcome it, we aim to support the development of more affordable energy conversion devices that use BPMs in the forward-bias mode.

## Supplementary Material



## Data Availability

Data will be made available on request.

## Notes

Data on the effect of the flow rate and the total concentration of electrolytes on the forward-bias performance, data on the reverse bias of Fumasep FBM in either “pure” or “contaminated” electrolytes, data on the BPM stress test under forward bias, and data on the performance of custom-built BPMs with various CEL thicknesses (PDF)
