# Supplementary material for: Salt Ion Accumulation in Bipolar Membranes Limits the Maximum Rate of Neutralization
Source: ACS Appl Mater Interfaces. 2025 Jul 29;17(32):45713–21. doi: 10.1021/acsami.5c08661 (PMC12356540; doi:10.1021/acsami.5c08661)
Supplement: Supplementary file 1 [file am5c08661_si_001.pdf]

Supporting information

Salt Ion Accumulation in Bipolar Membranes Limits the Maximum Rate of Neutralization

Pavel A. Loktionov[1], Erik M. Kelder[2] and David A. Vermaas[1]***

[1] Department of Chemical Engineering, Delft University of Technology, Van der Maasweg 9, 2629HZ, Delft, The Netherlands

[2] Department of Radiation Science & Technology, Delft University of Technology, Mekelweg 15, 2629 JB, Delft, The Netherlands

* corresponding author: p.a.l.loktionov@tudelft.nl

** corresponding author: d.a.vermaas@tudelft.nl

Keywords: bipolar membranes, forward bias, ion exchange, mass transport, neutralization reaction

1. Additional data

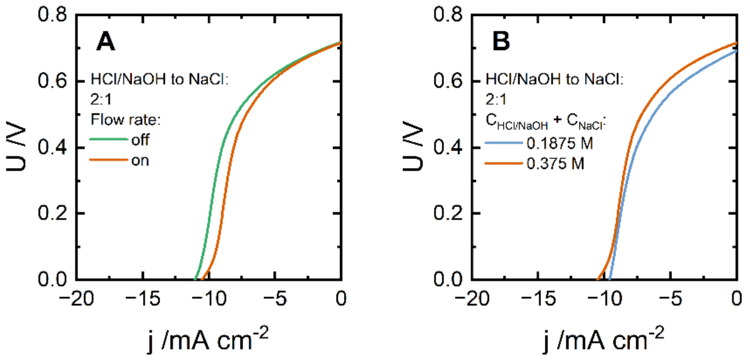

***Figure S 1**. Variations in flow rate (A) and total concentration of electrolytes (B) for forward bias in Fumasep FBM, which uses NaCl-based electrolytes.*

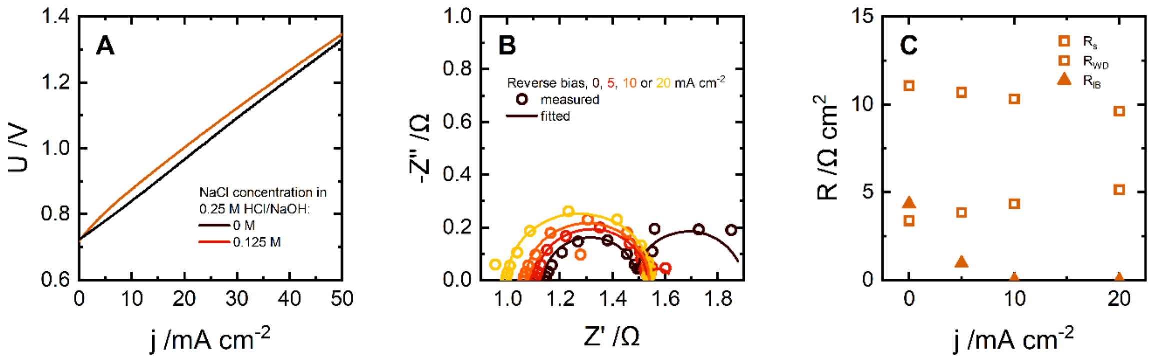

***Figure S 2**. Reverse bias of Fumasep FBM, which uses either "pure" or "contaminated" electrolytes (A) data on electrochemical impedance spectroscopy for the latter case (B), and resistance breakdown of impedance spectra shown in panel B (C).*

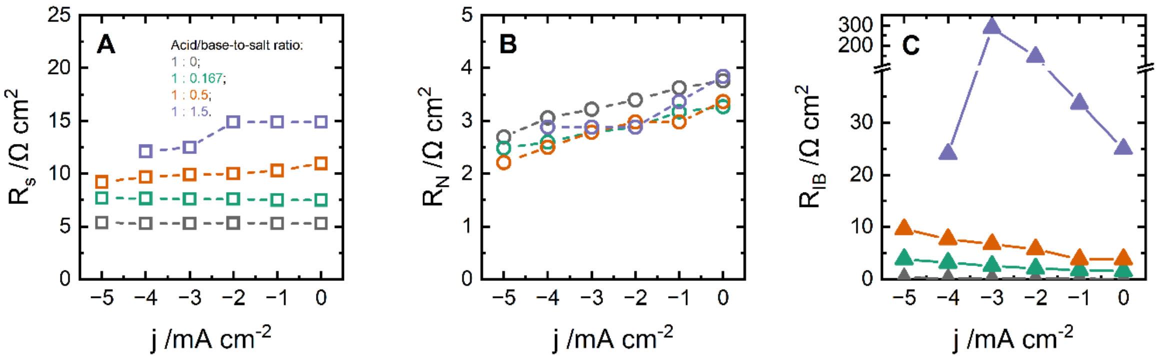

***Figure S 3**.Analysis of impedance spectra data of Fumasep FBM, which uses electrolytes with various acid/base-to-salt ratios; figure shows full dataset partially presented in Figure 3 of the main text.*

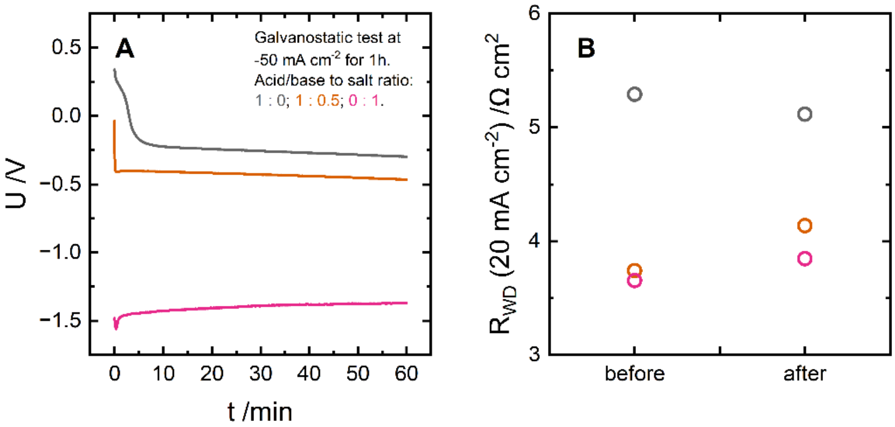

Figure S 4. *Transients of Fumasep FBM potential during stress tests under forward bias at -50 mA cm^{-2} (A) and change in resistance for water dissociation (R_{WD}) after the tests extracted from impedance spectra recorded under reverse bias at 20 mA cm^{-2} (B).*

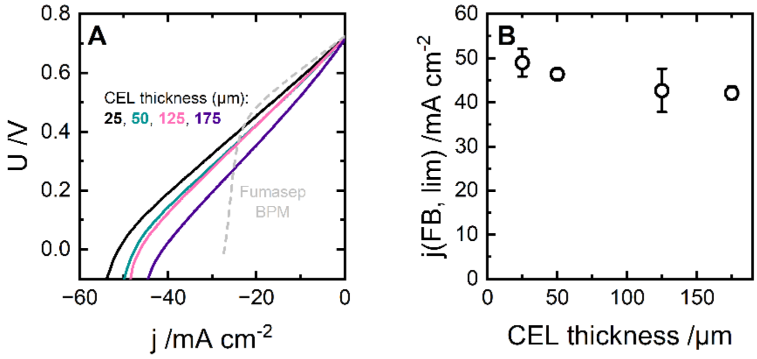

Figure S 5. *Forward bias polarisation curves of custom-built BPMs (A), which use salt-contaminated acid solution and dependence of the limiting current on CEL thickness (B); CEL is Nafion 211, 212, 115 or 117 (25, 50, 125 or 175 µm), AEL is Piperion 20 (20 µm), catalyst – GOx (9 µg(GOx) cm^{-2}); electrolytes: 0.25 M HCl + 0.125 M NaCl, 0.25 M NaOH; panel B includes average values of j(FB, lim) with indication of standard deviation based on two membrane samples; data for Fumasep BPM included for the reference.*
